# Green Care Farms as an Approach to Support People Living with Dementia: An Exploratory Study of Stakeholder Perspectives

**DOI:** 10.3390/ijerph22071016

**Published:** 2025-06-26

**Authors:** Anthea Innes, Vanina Dal Bello-Haas, Equity Burke, Rebekah Churchyard, Ingrid Waldron

**Affiliations:** 1 Gilbrea Centre for Studies in Aging, Faculty of Social Sciences, McMaster University, Main Street West, Hamilton, ON L8S 4M4, Canada; burkee3@mcmaster.ca; 2 Centre for Rural Health Science, University of the Highlands and Islands, UHI House, Old Perth Road, Inverness IV2 3JH, UK; 3 School of Rehabilitation Science, Faculty of Health Sciences, McMaster University Main Street West, Hamilton, ON L8S 4M4, Canada; vdalbel@mcmaster.ca; 4 Green Care Farms Inc., 9365 10 Side Rd, Milton, ON L9T 2X9, Canada; rebekah@carefarmscanada.com; 5 Department of History, Faculty of Humanities, McMaster University, Main Street West, Hamilton, ON L8S 4M4, Canada; waldroni@mcmaster.ca

**Keywords:** green care farms, dementia, benefits, physical health, social health, mental health, day care provision, green care, farm

## Abstract

How to best support people living with dementia and their care partners living in the community to maximize their quality of life and quality of living through appropriate and effective non-pharmaceutical approaches remains a focus of dementia societies and organizations worldwide. This paper examines the views of a range of stakeholders about the potential of green care farms in Canada, a country new to the concept of the green care farm approach to dementia support and care. Data were collected in Southern Ontario, Canada, between June and August 2022 via an online questionnaire (*n* = 12) and 1-1 interviews (*n* = 6). Questionnaire data were analyzed using descriptive statistics, specifically counts and frequencies. All interviews were audio-recorded and fully transcribed verbatim and analyzed thematically. We report thematic findings relating to the understanding of care farms for people living with dementia, perceived benefits of care farming, perceived enablers and barriers to implementing such an approach, and the hopes, motivations, and expectations of different stakeholders. The potential of green care farming for people living with dementia and their care partners in the Canadian context was evident. There are implications for care policy and practice relating to the promotion of (social) health and wellbeing for people living with dementia.

## 1. Introduction

There are more than 55 million people living with dementia globally, with the numbers projected to rise further [1]. Common consequences of dementia include loneliness and isolation [2] and the loss of physical function and mobility [3]. Those living in rural areas are likely to face rurality-specific issues compounding impacts to mobility from poor public transportation, gaps in digital connectivity, and decreased social connection contributing to increased loneliness and isolation further exacerbated with dementia [4]. As people age in rural communities, they may face challenges in maintaining their independence and experience a reduction in social and physical mobility [4]. Policy directives [5] reflect growing concerns about how to best support community-dwelling people living with dementia as the numbers with the condition are projected to increase.

Previous research has found that while rural- and remote-dwelling people with dementia do participate in a variety of pleasurable activities to maintain psychological health, participation rates for physical activity and engaging in the recommended amounts of weekly exercise are much lower [6]. Furthermore, individuals living with dementia were less likely to believe in the importance of physical activity and exercise for health promotion [7]. Yet, it is important to note that other research reports people with dementia living in rural areas having a preference for being outdoors and keeping physically active with everyday tasks [8]. The importance of being in nature and connecting with the natural world has been examined by a recent review [9], demonstrating the growing recognition of the value of being in nature.

What has become known as the care farm approach to providing support to people living with dementia, as a day program or as residential care, has been applied in other countries, for example, the Netherlands, Norway, Italy, Germany, Japan, and the United Kingdom, for many years [10]. Care farming may involve a range of outdoor activities designed to encourage physical activity and mobility, such as working with and feeding animals, farm-related work (painting fences, horticulture (defined as the art and science of growing fruits, vegetables, flowers, trees, shrubs, and ornamental plants), and gardening (this can be simple everyday gardening tasks, such as cutting the grass and weeding)), walking, and social mobility, including participation (this can be a one-off event), community engagement (normally ongoing), and socialization (in-the-moment social interactions) [11,12,13,14].

A recent scoping review [15] explored the types, opportunities, benefits, and challenges of outdoor-based care and support programs, including care farms, for community-dwelling people living with dementia and their care partners. Care farms offer a unique way to support people living with dementia using accessible rural outdoor spaces to promote physical, social, and mental health and wellbeing via involvement in outdoor activities [16]. A variety of benefits have been reported from day programming, including but not limited to the following: (1) increased physical activity and physical effort levels [17,18,19,20,21]; (2) increased mood and emotional wellbeing [17,18]; (3) increased quality of life [20]; (4) fewer behavior problems and less need for medical or other healthcare interventions [19]; (5) enhanced sense of belonging [15,20]; and (6) care partner benefits, such as respite and decreased care burden [15,22].

Though literature exists on the benefits of care farming from other countries [14], there is a lack of application in the Canadian context, a gap this exploratory research aimed to address.

## 2. Materials and Methods

This multi-method, exploratory, descriptive research aimed to address the following: (1) the perspectives and views of different stakeholders on the potential of green care farm programs for those living with dementia in Canada. The care farm program under consideration was for a day program to be situated on an existing “pick your own” fruit/vegetable/flower farm. Two methods of consultation were selected. The first was an online questionnaire for those interested in volunteering at a care farm (e.g., administrative tasks such as advertising, social media, and recruiting potential participants, or helping set up the physical landscape of the care farm using gardening, horticulture, or construction skills) that was being developed. The second approach, 1-1 interviews, was to gain a deeper insight into the perspectives of those who would be more involved in the day-to-day activities of the care farm, including the farm location staff and those who would be responsible for delivering the care farm to people living with dementia and a care partner and person living with dementia.

### 2.1. Participants

Our convenience sample of participants (*n* = 18) were Ontario-based stakeholders who were interested in contributing to developing a green care farm in Canada and included the following: a person living with dementia and their care partner; farm staff from a farm with the potential to be a site for a green care farm program; and individuals who would be willing to be responsible for the delivery of a green care farm program, including administratively or on a volunteer basis.

### 2.2. Online Questionnaire

The questionnaire was developed and reviewed by researchers and a community stakeholder prior to its distribution.

The online questionnaire link was sent to 35 community volunteers identified through an organization considering setting up a green care farm program during the summer of 2022 in a rural area of Southern Ontario. The online questionnaire was active from June to August 2022, and one reminder email was sent. The questionnaire (Appendix A) included 10 questions that provided volunteers the opportunity to anonymously share their experiences of volunteering, motivations, and expectations for joining a care farm initiative designed for people living with dementia.

### 2.3. Interviews

Semi-structured interviews were selected to encourage a purposive sample of stakeholder participants *(n* = 6) with lived experience of dementia, dementia care experience, and farm-based experience to speak in-depth about their experiences, expectations, and perceptions of the new concept of a care farm model of support for people living with dementia. A topic guide was utilized in all of the interviews (see Appendix B) to facilitate consistency and was designed solely for the purpose of the study. The topic guide was used to guide the interviews to ensure that certain topics were covered but was not used to stifle or suppress information that the participants felt was meaningful, thus allowing new topics to emerge. The interviews were about 60 min in length, and all interviews were digitally recorded, transcribed verbatim, and anonymized by assigning each participant a code number, P1 to P6.

### 2.4. Data Analysis

The survey data were analyzed using descriptive statistics, specifically counts and frequencies. Open-ended comments were thematically analyzed alongside the interview data. The interview data were analyzed using an inductive thematic approach [23]. The main aim of this thematic analysis approach was to identify patterns within the data, which could then be defined as themes. The initial step within the process of a qualitative data analysis is for the researcher to familiarize themselves with the data. All of the audio files from the interviews were listened to, and the transcripts were read. A particular focus of this process was to consider the research questions whilst listening to the data, and notes were made to provide a preliminary overview. Data were initially coded by one researcher, and the data were categorized into groups of meaningful quotes. More structured codes and themes were then developed by three researchers. The process of the thematic analysis was iterative and involved the researchers regularly engaging with the data to interpret what was emerging by coding the data, generating themes that were then discussed, amended as required, and agreed upon following five iterations. The themes presented below that emerged from the data coding and analysis process at each phase of analysis were then compared to provide a triangulation of the data from each source. This allows for a holistic understanding of the study findings and strengthens the trustworthiness of the findings and conclusions.

## 3. Results

We first report on the analysis of the online questionnaire completed by those indicating a willingness to volunteer their time for a new green care farm initiative. We then provide an analysis of the interviews conducted with other stakeholders and discuss the thematic issues emerging from our analysis.

Of the 35 individuals invited to complete the online questionnaire, 12 participated (34.2% response rate). Of the 12 participants, 4 (33.3%) partially completed the questionnaire (e.g., left some questions unanswered), and 8 (66.6%) fully completed the questionnaire.

### 3.1. Questionnaire Findings

The participants were asked about roles held and tasks undertaken. In relation to the volunteer role(s) previously held by those who responded (66%), five had held on-site community day volunteer roles, one had an administrative role, and two had both on-site and administrative roles. When asked about the tasks completed in the volunteer role, two described gardening roles such as planting and building raised beds, and one described a cleanup role of removing dangerous objects from the land. Three participants described their tasks as strategic planning, support, and fundraising. All participants stated that they would be willing to volunteer again, reflecting a commitment to community altruistic endeavors. When asked what would incentivize them to volunteer, the responses were all individual and included the freedom to come when available, being outside and involved in activities with the community, and volunteering in a position that would help individuals. Some outlined incentives related to individual benefits, including one participant who responded using their skills and knowledge and another who prioritized having a choice of activity that suited them. Others mentioned the satisfaction of helping others. When asked about any support that they might require to volunteer, three participants felt that there was no additional support that they could think of that would enable them to participate in the future. One stated that they would require transportation to and from the venue of a green care farm to participate in the future. One responded that they would require being heard to make sure that it is also still fun and worthwhile to them. This was an interesting response in that it could be showcasing someone’s desire to be more involved in planning or to be consulted to ensure that it is still something they want to pursue. Finally, one respondent asked for more support at home to enable them to volunteer.

When asked about how the participants had heard about the opportunity to volunteer, the primary form of being recruited to volunteer was through personal contacts and word of mouth. This provides insight into the value of relationship building to develop a volunteer cohort to support care farm activities.

The participants reported multiple reasons (between 4 and 6) about their different interests and motivations to volunteer on a green care farm. Half *(n* = 6) of the respondents reported the opportunity to work outdoors, one-third (*n* = 4) reported their motivation as previous experience of a family member with dementia, and a further two (16%) reported previous experience of dementia care. Additionally, 25% *(n* = 3) wanted to contribute to their local community, and one-third *(n* = 4) wanted to use their skills (for example, horticulture, gardening, and visualization). The most popular response was an interest in the concept of care farming (58%, *n* = 7).

When asked about their understanding of care farming, seven participants provided responses. The responses varied but had similarities among themes such as inclusion, meaning, purpose, enjoyment, and participation in activities for clients living with dementia. Two reflected on safety and supervised activities, while others (two respondents) spoke more generally of care farming as the act of engaging in light gardening in an outdoor environment. Others (four participants) referred to the therapeutic aspects of care farming, including the mental, physical, and social impacts of engaging with nature and learning new skills for those living with dementia. One response made a comparison to traditional indoor programs that the participant felt lacked some of the significant mental and physical health benefits found in care farming for those living with dementia.

Respite was the most commonly reported benefit for care partners *(n* = 6) when asked about perceived benefits of care farms. The perceived benefits for the person living with dementia varied; one spoke about the satisfaction and accomplishment that those involved in the program could feel through the farming progress and engagement in familiar activities, two participants explored the idea of bonding and socialization through the opportunity to connect with others in the program, and two respondents discussed the mental stimulation and cognitive benefits that the care farm program could have in potentially delaying the progression of dementia by engaging in purposeful and familiar tasks such as those in the program.

Based on the responses above, volunteers who were interested in supporting a care farm program for people living with dementia could see the potential benefits of the program based on their initial expectations, their understanding of what care farming is, and the impacts that the care farm could have on the wellbeing of people living with dementia, their families, and care partners.

### 3.2. Stakeholder Interviews

We now present our findings from the stakeholder interviews. The participants included the following: a person living with dementia, a care partner, and four stakeholders with a dementia care background or a farming background. To be compliant with our ethics approval for this study, specifically the need to maintain the anonymity and protect the identity of the participants, no demographic data are reported. Our four primary interconnected themes, informed by a social inclusion perspective, will now be discussed.

#### 3.2.1. Theme 1: Understanding of Care Farming

The participants perceived care farming to offer an alternative to traditional forms of care with the opportunities to engage and connect with nature and the seasons. The participants also felt that this care model would allow for the opportunity to retain interests and associated skills in participating in farming and/or gardening-based activities.

##### Alternative to Traditional Forms of Care

The participants clearly articulated the potential client group for care farming as an alternative to traditional indoor day care style programs:

*“..to serve these older adults with dementia who struggle with traditional programming”* (P2)

*“yeah, but you’re providing care in a natural environment, this is nature—it’s not a hospital”* (P3)

Care farms provided the opportunity to perform different things that clients of a day care program may not be able to do and, importantly, to do those activities outside:

*“So, that the providing them with multiple opportunities for them to do stuff—just anything really and that’s the nice thing about a farm, is, there’s tons of different things to do”* (P3)

*“My hope is that people with dementia will have access and engagement with the outdoors with lots and lots of living things agriculturally, so animals, horticulture—really plants”* (P1)

Care farms were, therefore, perceived to be an alternative place to provide support and care to people living with dementia in a setting promoting the opportunity for different types of activities outdoors.

##### Engage and Connect with Nature and the Seasons

Related to the alternative mode of care delivery was the perception of the care farm location to promote engagement and connection with nature and the seasons.

*“breathing in the fresh air, you have somebody walked by and say hey check it out, we just pick some cherries, you know there’s again opportunity for relationship and engagement, (P3)… Maybe sitting in the shade, maybe they’d love to be sitting under the cherry tree for five hours, instead of sitting in the room for five hours, you know I think I’d rather sit under a cherry tree”* (P3)

The potential for the outdoor space to become both familiar and empowering was also discussed as follows:

*“the garden space and the land, and this becomes empowering to them in a way too, and becomes—feels a bit like home”* (P2)

With participants recognizing the healing potential of being outdoors:

*“I spend a lot of time in the garden, and I know what it’s done to me in a healing sense, it got me through a difficult period of time”* (P3)

And how this connected with promoting a healthy lifestyle:

*“…and give them an opportunity to be outdoors and really connect with these elements of a healthy lifestyle”* (P2)

The recognition of the mental health benefits of being outdoors and creating the opportunity to do so for people living with dementia was, therefore, an important perceived benefit.

##### Retain Interests and Associated Skills

The participants recognized that the opportunity to attend a care farm would enable people living with dementia to retain previous interests and skills:

*“appealing to those people with Alzheimer’s and dementia who have a background or history, some interest in farming and gardening, and connecting with their interests and familiarity”* (P2)

And that this could provide a sense of purpose beyond the enjoyment of the “in the moment” participation in outdoor-based activities:

*“have an opportunity there to develop, like you’re participating in something. Like growing food for the food bank say. It’s that purpose right, that’s not just a past time”* (P3)

The participants’ understanding of care farms related, therefore, to the following: perceptions of the appeal of this kind of alternative care program compared to traditional day care models; the opportunity outdoor-based care had to provide connections with nature, the seasons, and other people while also creating a space for individuals with a prior interest in farming or gardening to come together and use their skills; and ways that could go beyond the immediate enjoyment of gardening to a sense of purpose when growing produce that could be enjoyed by others in need.

#### 3.2.2. Theme 2: Perceived Benefits of Care Farming

The participants perceived three primary ways that care farming could bring benefit to the person living with dementia and, indeed, benefits for all involved in a care farm initiative, for example, volunteers, staff at the farm, and staff supporting individuals within the care farm setting. The perceived benefits fall into three interconnected sub-themes: belonging, purpose, and the promotion of health and wellbeing.

##### Belonging

The intention of the care farm was to promote a sense of belonging and meaningful engagement for people living with dementia:

*“so people with dementia will engage in activities where they feel a sense of belonging, they feel a sense of responsibility And purpose, it’s not busy work, it’s not entertainment, [it’s] that what they’re doing matters”* (P1)

Joining an initiative designed to bring people living with dementia to achieve a common purpose in a farm setting was perceived to offer not only a sense of belonging but also a sense of purpose:

*“…to promote purpose and belonging for people with dementia”* (P3)

But this sense of belonging was perceived to extend beyond benefiting only the person living with dementia:

*“helping each other out right, and working with, again, with other clients [people living with dementia] and with other staff and with volunteers to like, it’s just there’s so many different types of people who can participate in the program. It’s not just you know, the clients, it’s a lot of different types of people and so everyone can be engaged in something purposeful… working together to try and get food grown and get it out, you know, and harvested”* (P3)

Importantly, the farm environment was perceived to promote working with rather than caring for (in the traditional sense of task-based care) the person living with dementia:

*“you’re working with clients, you’re not caring for clients… you’re caring for them, but you’re doing it with them so you’re planting things with them you’re not just taking care of them, right”* (P3)

The importance of working alongside the person living with dementia rather than “doing” things for or to them is an important distinction in how green care farms were perceived.

##### Purpose

Having a sense of purpose was an important perceived benefit of care farming for people living with dementia. This is related to working with nature in the physical location of the farm to grow produce:

*“I think that combination of providing the space on the farm here and growing food, but then also working with this program where there is support staff for those people with dementia who have different needs and abilities, that they have that support to really engage in a meaningful way in the space and on the farm”* (P2)

Linked to this was the benefit that could arise from the sense of purpose of growing food to benefit and help others:

*“Just to help …. To donate to some place... to donate the vegetables to a food drive or something ….”* (P5)

Care farming was seen by our participants to provide a conduit to promote a sense of belonging and purpose, both of which are key aspects of health and wellbeing [22], the final subtheme that we will now discuss.

##### Promotion of Health and Wellbeing

The benefits of staying physically, socially, and mentally active when one has dementia are well known [24,25], and care farms were seen to promote this kind of activity:

*“And just the benefits of being socially and physically engaged, like yeah, those have its own quality of life benefits …”* (P1)

With mental stimulation perceived to be a benefit:

*“I think anytime, especially with dementia, I think when you can wake up a memory… that fascinates me, how it’s just something from so long ago, [farm skills] I think it’s cool. Again, seeing somebody light up [when their memories/skills come to the fore]…”* (P4)

The opportunity to promote social wellbeing, in particular, was stressed by the participants:

*“The sociability [of care farming] is amazing…”* (P6)

*“I think that it’s really important for mental health to still feel connected. And part of that connection is being able to joke around with people, and a little bit of getting to know each other”* (P3)

The benefit to health and wellbeing derived from a sense of accomplishment from growing produce was also highlighted:

*“See, “see what I’ve accomplished.” That’s good”.* (P6)

And the joy of being in nature and the sense of wellbeing that this can engender:

*“and there is something very joyful about holding the flower, or smelling a freshly cut hayfield, like there’s just these like things that we don’t have good language to explain, there’s this—the sensory engagement”* (P1)

The perceived benefits of care farming demonstrate the opportunities such a care approach can bring to a person living with dementia through joining others and feeling a sense of belonging to a group working together to grow and harvest produce that creates a sense of meaningful engagement through purposeful activity, which, in turn, promotes health and wellbeing. As such, the benefits of care farms extend beyond the primary client group, in this case people living with dementia, to the range of other people who work together with those living with dementia on the farm.

#### 3.2.3. Theme 3: Perceived Enablers and Barriers

The participants discussed various enablers and barriers related to the design and implementation of care farming. Enablers and barriers were framed within the context of the overall importance of care farming for people with dementia.

##### Enablers

The perceived benefits of care farming cited by the participants and described above could be considered enablers. Having additional resources and supports was discussed as a way to allow for different care farm program approaches and models to be applied.

*“Yeah, I would like that too, that’s not what this model is right now, but that would be so cool if we could pay people with dementia to work and pay the farmer, the question is who is paying for it right, so.”* (P1)

A “value add” enabler that was described was having a care farm situated on a working farm. This specific program design element was seen as having mutual benefits.

*“We loved the idea at the farm here, and we happened to have this space here that we’re standing at now that’s about a quarter acre in size. So, it’s a good size. We can grow a lot of food here, but it’s not so big that it’s overwhelming … Care farm program here was exciting to us as well, and it, yes, really activates and makes this space exciting on the farm here.”* (P2)

*“The idea of more people on this farm doing something….They’re bringing more people to the knowledge of just how hard we work but they may never have that exposure … It’s like the perfect little spot and it needed something. This is just like it’s a fit…. We can cover what we can do because it also helps our farm.”* (P4)

The situated nature of care farming was also viewed as a way to engage with the larger community, specifically older adults, and as an opportunity to educate the community about the importance of farms and farming to the community.

*“I think one of the—perhaps one of the missing pieces of the farm here is how do we engage that older demographic in the community as well and ensure that not only kids and families are connecting with the farm, food, and the land, but there’s also an opportunity for elderly people in our community or people with different needs and abilities, have the opportunity to also continue coming back here and feel they have a place here to connect with what’s happening.”* (P2)

*“We can eventually get the rural, get the local community [to] realize just how important we are.”* (P4)

*“So, that part of how we farm is very exciting to us is how we invite the public here; there’s not many farms that do this; learn about how food grows, taste food fresh out of the field.”* (P2)

Having a care farm program situated in the natural environment of a working farm was viewed as a highly desirable feature. This non-traditional, nature-based partnership was considered a springboard for drawing in the community to the working farm and increasing awareness about farm-to-market-to-table.

###### Barriers

Regardless of their interest in care farming, the participants cited risk as a barrier. Care farms being situated in a natural environment, a working farm, brought with it risk considerations. Risk was often described as safety concerns for the person living with dementia and their participation in the activities on the care farm. Risk was also specifically identified as concerns about potential liability issues by several participants. Liability-related barriers were not necessarily “quick fixes” or amenable to “fixing”, e.g., weather, farm environment. Liability-related risks that could be mitigated would require further investigation and strategizing and additional resources and support. For example, one participant stated the following:

*“So, that’s something we all worry a little bit about, liability or what if somebody falls and hurt themselves, or what if they—the heat is too extreme and they—[Laughter] and this is why, as the farm, it makes us feel more comfortable we know there’s a support worker there … We’re not wheelchair accessible at this time, the washrooms are a little bit of a walk away, we haven’t got a lot of protected space if there’s intense wind or rain or something.”* (P2)

*“yeah I, I think the big one that sticks out to me, is when you get more than one person on site okay, and you start to have people with varying degrees of capabilities right, and varying interests varying intentions.”* (P3)

While risk was largely considered as concerns about safety and liability, risk was also related to determining or navigating the “trade-off” between safety and liability as risk versus maintenance of agency and personhood for the person living with dementia. Risk was of concern to participants, not only in relation to the liability that comes with the responsibility of providing this form of care support, which poses a potential physical risk of harm, but also the stigma dementia brings and the perceived risk of accessing a farm setting for a person living with dementia.

*“Yes, because I think when people start the beginning of dementia and all that, I think people lock them down. Then, they don’t know how to let go.”* (P4)

Potential difficulties recruiting people living with dementia into care farming were noted. Determining the best methods for recruitment in consideration of the potential population being drawn from and how to best frame the program to assist with recruitment were discussed.

*“It’s not a demographic that’s comfortable on the computer. So, how do you get to them? …. That’s challenging in terms of the demographics and the computer and who you’re reaching. Are you catching the caregiver and their needs and what they want for their loved ones?”* (P4)

*“so that’s a big challenge …. getting the word out like that one super tricky and a lot of our nonprofit friends have … it’s hard for them to endorse a for profit model … need to get the word out, and those are the communication and distribution channels that people receive information, most of the times… An opportunity, but I guess a challenge, to message the program to figure out what the messaging is because … (it’s) not a health care service.”* (P1)

Views on the cost for participants to attend the care farm varied depending on the participant and their involvement in the program. For example, questions that arose included the following: Should this be a free service? How can it be a viable business/social enterprise model? What would be the financial return for any farm hosting such a care approach?

*“Okay, my personal view is that healthcare and healthcare related things should be free. Free access, because their basic human needs…. It shouldn’t have to cost money to get out and be in nature, and yet when you have high care needs it does cost money, because you need somebody to be with you, right…* (P3)

Some of the participants discussed whether not having formalized transportation access as part of care farm programming could be a barrier to attendance and participation. In other words, could offering transportation as part of care farming program models facilitate access and assist with recruitment? Transportation was not being offered as the program was in its infancy and carried liability considerations.

*“Maybe you offer bussing [to enable participants to get to the care farm]”.* (P4)

Having a program for people living with dementia in a natural physical environment, as opposed to a more typical dementia care environment, was viewed as a facilitator of engagement—not only for the person living with dementia and the care farm but for the working farm as well. The barriers were mainly centered around the potential hazards associated with care farming being situated in an environment that is open to the elements. Identifying liability, risks, and safety issues, as well as how to best manage these issues, was discussed by all participants. Concerns about liability further hindered considerations of offering transportation, which could facilitate access for persons living with dementia.

#### 3.2.4. Theme 4: Motivations, Expectations, and Hopes

Throughout the interviews, participants explored their motivations for becoming involved in care farming, the expectations they had about the impact of the program, and the hopes that they held for the program. This theme was raised in every interview with the participants, though in different contexts, but all speaking to the importance of care farms to answer why they got involved, what they anticipated, and what the future may hold for these programs.

##### Motivations

Most of the participants, regardless of the reasons underpinning interest in care farming, shared the main motivation for becoming involved as giving back, volunteering, and helping others. This included, for example, helping those living with dementia, helping with farm work, and donating the food grown back to the local food bank.

*“I just I thought I would be able to come and help somebody”* (P5)

*“…have an opportunity there to develop, like you’re participating in something. Like growing food for the food bank say. Its that’s purpose right, that’s not just a past time”* (P3)

*“That they would be growing these [produce] and then they would be giving them [vegetables] to others that are in need”* (P6)

Another common motivation was believing in the vision and mission of the program and its impact on older adults living with dementia and their families. The unique design of the program resonated with the participants.

*“…it just seemed like wow! what a huge, what a beautiful opportunity.”* (P3)

Many acknowledged the current gap in care for those living with dementia and the role that care farms could play in filling this gap that contributed to their desire to take part.

*“…hearing from a lot of older people just really kind of solidified, Okay, we need this and society that’s pretty clear to me, we don’t have enough people working on this who are interested in it”* (P1)

*“Just from my understanding, it’s much more common or well-established to have these kinds of programs in Europe and other places of the world, but to educate more about the need for these kinds of programs here [Canada] and then see that support build and partnerships expanded to make it happen on a, I’m, going to say broader scale.”* (P2)

Many of the motivations or underlying reasons for inclusion in the program by the various stakeholders were based on the high expectations or aspirations that they felt this model of care and support offered.

##### Expectations

The intended goal for the program was to increase the wellbeing of people living with dementia. Many shared the expected benefits to the physical, mental, and social wellbeing of older adults by participating in care farming.

Many articulated the expectation that the program should benefit the wellbeing of not only the person living with dementia but also their families and care partners by offering some respite while their loved one was at the program.

*“I always felt I didn’t have to worry … He would be looked after and he would be doing something he likes to do.”* (P6)

Many anticipated that the program would offer a unique social opportunity to engage with staff, clients, and their families in a different, more personalized way than traditional care programs/services.

*“ That care and attention is there to the needs of the participants when they’re actually in the site so that gives us more comfort both for the program and for the plan”* (P2)

Participants often aligned their motivations and expectations for the program with the underlying hopes they have for a care farm model of support for those living with dementia.

##### Hopes

Given the newness of the care farm concept in Canada, many explained their hope for it to grow through increased awareness of the model and more information on how to get involved. This would ultimately increase the number of people impacted by the benefits of the program, as well as the benefit of peer connection among those in the program.

*“I really do hope it’ll get off the ground. … it’s getting people to understand that it is helpful and it is good for social. That is, I think, really important.”* (P6)

Along the lines of growth, some hoped the program would develop into one offered all year round, with varied activities.

*“I am hoping it will be on five days a week”* (P4)

With growth comes recognition and the desire to have care farming’s importance recognized on a global scale and utilized as a pilot or prototype for similar programs.

*“It would be very exciting if this idea can be looked at as something—as an example of what could be done at other farms throughout Ontario, throughout Canada”* (P2)

The motivations, expectations, and hopes shared by the participants showcase the importance of care farming directly from the perspective of those on the front lines. It provides an insightful look into what contributes to a successful program, and results could be applied to future programs as a rationale or example in practice.

## 4. Discussion

The participants (both questionnaire respondents and stakeholder interviewees) perceived care farming to offer an alternative to traditional forms of care with the opportunities to engage and connect with nature and the seasons. This was seen as a real opportunity to promote health and wellbeing by working outdoors and using or developing gardening-based skills [12] and reflects the benefits experienced by other countries, for example, the Netherlands [10,22] and Norway [17,20], who have implemented care farms into their portfolio of care provision. Part of the appeal for participants was that it was perceived as a different way to provide support than traditional models of day care. There were many perceived benefits to not only the wellbeing of the person living with dementia but also for all who may become involved in care farming. These included the following: connecting with nature, connecting with others, promoting a sense of belonging, and also purpose via meaningful activity, all of which would contribute positively to social health and wellbeing [11], one of the key objectives of quality dementia care provision [24]. In addition, creating the opportunity to be outdoors is something that can be denied to people living with dementia whose social worlds shrink [25], as do the physical “safe zones” people living with dementia venture out to explore [26].

The participants discussed both enablers and barriers related to designing and implementing a care farm model of support, and these aligned with their perceived understanding of care farming and their hopes for future developments. Guides on supporting rural-dwelling people living with dementia are useful here [4], as they point to the importance of providing outdoor-based opportunities for people who have extensive connections with the land. Also, practical considerations of risk and liability, transport, and costs were flagged by the participants. Safety concerns about mobility have been previously considered by others thinking about how to best support people outdoors and demonstrate that it is possible to work within safety concerns [15,21] and a need for risk tolerance as part of the informed consent process when choosing to take part in such initiatives. In addition, transport to care farm locations needs to be considered, particularly in areas where public transport is less available.

The participants’ expectations of the care farm and their views on how it could be developed into a routine form of care support varied, reflecting the newness of the concept in the Canadian context. It was acknowledged by many that the lack of care farming initiatives in Canada pointed to the gap in available services, and their hopes and expectations for current and future programming were dependent on growth in acknowledgment, value, and support in the form of funding. The biggest takeaway for many participants was the belief in the care farm initiative’s benefits and the potential for a new program to act as a prototype or exemplar of that for future programs. This belief was one of the strong indicators for volunteers’ motivation to be involved in care farming.

Applying stakeholders’ perspectives to practice development is important [8]. For example, our findings demonstrate that additional considerations may be required to determine the best method of recruiting workers (volunteers) and also people living with dementia into any planned care farm program. Further consultation with the local population where a care farm is to be located is important to see what they would believe would work in practice, e.g., how do older people find out about an initiative, and how do they feel confident to begin attending? Recruitment through non-traditional 1:1 communication (rather than online ads or social media) due to the unique nature of care farms requires more intimate conversations to allow for a clear understanding of the program and aims. In-person outreach or partnerships with community organizations to improve accessibility and participation could be beneficial to recruit people living with dementia to join care farming programs.

How to promote awareness of the program to the intended beneficiaries is key. Raising awareness, as well as confidence to attend, could be promoted via informal visits and open days to a proposed site to enable potential clients (a person living with dementia or the care partner) to become familiar with both the setting and the program.

Developing a care farm model requires consideration about the location of the land or farm where the program will be operated, as well as specific jurisdictional elements that may affect funding and reimbursement for this care and support. The benefits of establishing a care farm on a well-developed wider farm are many, such as having access to resources, farming knowledge, and operational support, as well as access to other community members, building on the reputation of the farm, and shared liability [18]. Other related benefits of working with an existing farm relate to the permanency and legitimacy of the activity versus setting up a “for purpose” site. Care farming has been established in Norway [17,20] and the Netherlands [9,21] for many years. Programs offered in these countries combine different agricultural activities on a continuous basis, such as growing crops and managing livestock, with care and support services, e.g., adult day services and 24-hour nursing, as an alternative model of care for people living with dementia. The extent to which these types of models or elements of these models can be implemented and remain viable as a healthcare or business model in the Canadian context remains largely unknown. As does the potential for this kind of program to work within existing government funding and community models. The costs and who bears them and how this relates to the benefits of being on a farm where the farmworkers help to support when the program is not running is an issue that would benefit from further research, as would an extension of the research to date to obtain greater insight into stakeholder perspectives.

### Limitations

The primary limitations of this exploratory work are the small convenience sample, where only 12 of the 35 volunteers interested in care farms completed the questionnaire and only 6 stakeholders participated in an interview, as well as the limited wider applicability due to the specific context, e.g., southern Ontario, from which the sample was drawn. Other stakeholder groups, such as policymakers, funders of care, and others who were not already interested in the care farm approach, were not included in this exploratory work, and, therefore, the perspectives included in this research are limited. Future research could expand the sample size and location and use community outreach, for example, to recruit participants beyond the convenience sample included in this paper. Presenting the data in an aggregate preserves anonymity and confidentiality but limits detailed demographic data. Our small sample size may reflect the lack of implementation and knowledge about this approach in Canada.

## 5. Conclusions

Our exploratory research demonstrates an intuitive appeal of the care farm model for the stakeholders consulted, such as improved physical, social, and mental wellbeing through participation in outdoor activities that serve a purpose and promote social connections. However, further research is required as to how this would benefit Canadians living with dementia in practice. Future consideration is required as to how care farms might function within Canadian safety, regulatory, and cost frameworks. Overall, we found enthusiasm for a farm-based model of care, which highlights the need to rethink and reframe how services and programs are developed and implemented for people living with dementia in Canada. Future research could examine perspectives and understanding over time with participants who engage with the care farm concept in Canada. The findings may have relevance for Canadian provinces beyond Ontario and beyond Canada for any country where a care farm approach has yet to be introduced but which may be considered as an alternative way to provide support and care to those living with dementia. These findings could inform a wider study examining how lessons from care farms operational in other countries could be adapted in Canada. The concept of care farming would benefit from future comparative research to enable countries to learn from one another.

## Data Availability

Anonymized data may be available upon reasonable request but would need to be confirmed via the ethics board.

## Appendix A. Survey Questions

1. What volunteer role(s) have you held?
2. In your own words, what tasks have you completed in your volunteer role?
3. How did you hear about the opportunity to volunteer?
4. What motivated you to volunteer for care farming?
5. In your own words, what do you understand care farming to involve?
6. In your own words, what do you think the benefits to the person living with dementia (and their care partner/family members) might be of participating in a care farm program?
7. Would you volunteer to help support a care farm program?
8. In your own words, what would incentivize you to volunteer again?
9. Is there any support you feel you require to enable you to volunteer in the future?
10. Anything else you would like to comment on?

## Appendix B. Interview Topic Guide

1. How did you learn about care farming?
2. What interested you to get involved?
3. What are your hopes and expectations for care farms and those who get involved?
4. Do you anticipate any challenges or barriers in relation to care farms?
5. Do you have any concerns about care farms?
